# Graph Attention Network-Based Prediction of Drug-Gene Interactions of Signal Transducer and Activator of Transcription (STAT) Receptor in Periodontal Regeneration

**DOI:** 10.7759/cureus.68764

**Published:** 2024-09-06

**Authors:** Shubhangini Chatterjee, Pradeep Kumar Yadalam

**Affiliations:** 1 Department of Periodontics, Saveetha Dental College and Hospital, Saveetha Institute of Medical and Technical Sciences, Saveetha University, Chennai, IND

**Keywords:** drugs, genes, graph attention networks, periodontal inflammation, periodontal regeneration

## Abstract

Introduction

The signal transducer and activator of transcription-1 (STAT-1) are tightly controlled signaling pathways, with induced genes acting as positive and negative regulators. Persistent activation of the signal transducer and activator of transcription (STATs), particularly signal transducer and activator of transcription-3 (STAT-3) and signal transducer and activator of transcription-5 (STAT-5), is common in human tumors and cell lines. STAT molecules act as transcription factors, regulated by ligands like interferon-α (IFN-α), interferon-γ (IFN-γ), epidermal growth factor (EGF), platelet-derived growth factor (PDGF), interleukin-6 (IL-6) and interleukin-27 (IL-27). STAT-1 mutations can cause infections like periodontitis, a chronic inflammatory disease affecting gum tissue and bone. STAT-1 drug-gene interactions are being studied for therapeutic applications. Our study aims to predict drug-gene interactions of STAT-1 receptors in periodontal inflammation using graph attention networks (GATs).

Methodology

The study used a dataset of 215 drug-gene interactions to train and test a GAT model. The data was cleaned and normalized before being subjected to GATs using the Python library. Cytoscape and cytoHubba were used to visualize and analyze biological networks, including drug-gene interactome networks. The GAT model consisted of two graph attention layers, with the first layer producing eight features and the second layer aggregating outputs for binary classification. The model was trained using the Adam optimizer and CrossEntropyLoss function.

Results

The drug-gene interactome network, analyzed using Cytoscape, had 657 nodes, 1591 edges, and 4.755 neighbors. The predictive GAT model had low accuracy due to data availability and complexity.

Conclusion

The GAT model for drug-gene interactions in periodontal inflammation had low accuracy due to data limitations, complexity, and inability to capture all relevant features.

## Introduction

When interferons (IFNs) bind to specific receptors on the cell surface, they initiate a signaling cascade that activates Janus Kinases (JAKs). This activation, in turn, leads to the phosphorylation and activation of signal transducers and activators of transcription (STATs) [1]. Phosphorylated STATs form dimers and translocate to the nucleus, binding to specific elements in interferon-stimulated gene (ISG) promoters; thus they modulate gene expression and drive cellular responses. IFNs also induce the formation of other transcription factors. Cell-specific responses to IFNs are emphasized, with STATs -3 and STATs-5 being preferentially activated in human B-cells treated with Interferon-β (IFN-β). STATs -1 and STATs-3 have alpha and beta species due to differential splicing [2].

STAT is a tightly controlled signaling pathway, with induced genes acting as positive and negative regulators. Persistent activation of STATs, particularly STAT-3 and STAT-5, is common in human tumors and cell lines. Tyrosine kinases (TKs) are activated by binding extracellular ligands to their receptors, promoting uncontrolled growth and survival. STAT molecules act as transcription factors, regulated by ligands like IFNα, IFNγ, EGF, PDGF, IL-6, and IL-27. Type I interferons activate STAT-1 and STAT-2, forming dimers that regulate gene expression. It is known that STAT-1 mutations can cause common infections, such as periodontitis [1].

Periodontium, which supports teeth, consists of gingiva, cementum, periodontal ligament (PDL), and alveolar bone. Periodontitis is an inflammatory disease that causes degradation of these tissues, leading to tooth movement and loss. Current treatments focus on plaque removal and inflammation control but cannot fully restore the original tissues [3,4]. STAT-1 is a protein regulating the immune response. Studies have suggested that variations in the STAT-1 gene can influence susceptibility to periodontitis, as STAT-1 plays a role in the host's defense against bacterial invasion and inflammation. However, further research is needed to fully understand the relationship between periodontitis and STAT-1. One previous study reveals that STAT-1 is crucial in Nitric oxide synthase-3 (Nos-3) related hypertension and worsened periodontitis. It suggests it may be a key target for treating hypertension-related periodontitis, as it reduces proinflammatory cytokine expression and macrophage infiltration [1]. STAT, a transcription factor, directly transfers signals from cell membrane receptors to the nucleus, regulating gene expression. It's involved in cell proliferation, apoptosis, and tumorigenesis and is linked to various diseases. Activation of the JAK/STAT-1 pathway can upregulate IL-1 and transforming growth factor-b (TGF-b) expression in the kidney, making STAT-1 a potential therapeutic target for chronic kidney disease.

Predicting drug-gene associations of the STAT-1 receptor can help in treating periodontal disease by identifying potential therapeutic targets, promoting personalized medicine, aiding in drug discovery and repurposing efforts, and understanding disease mechanisms. By identifying genes and pathways involved in periodontal disease, new drugs can be developed or existing ones can be repurposed to treat the disease effectively. Understanding these associations can also provide insights into the underlying molecular mechanisms of periodontal disease, enabling the development of more effective treatments.

STAT-1 drug-gene interactions are being studied for potential therapeutic applications. Mutations in the STAT-1 gene can affect the response to IFN-α therapy, leading to impaired treatment. Other potential interactions include STAT-1 inhibitors targeting STAT-1 pathway components. GATs are neural network architectures for modeling and analyzing complex networks, such as drug-gene interactions. They are useful for tasks like node classification, where labels are predicted based on node attributes and relationships. GATs [5] use attention mechanisms to compute weights for edges, allowing the model to focus on relevant parts during learning. This approach helps researchers identify important drug-gene relationships, predict efficacy, and uncover novel drug targets, enabling more accurate predictions and targeted interventions in pharmacogenomics and precision medicine. Our study aims to predict drug-gene interactions of STAT-1 receptors in periodontal inflammation using graph attention networks (GATs).

## Materials and methods

Dataset preparation

Using probes and drugs [6], STAT receptor drug-gene interactions of 215 drug genes were downloaded and used for training and testing, with 80 percent and 20 percent data sets. The data underwent a cleaning process that involved the removal of outliers and normalization. This prepared data was then analyzed using GATs implemented through a Python library. In this network, drugs and gene names were represented as nodes, while target types served as edges. The activity_biochemical parameter was used as an edge weight, along with other relevant features, with the target_type also considered as the target for the analysis.

Cytoscape and cytohubba

Cytoscape is an open-source software platform for visualizing and analyzing biological networks, including protein-protein interactions (PPIs) and gene regulatory networks. It offers various features for network visualization, data integration, analysis, and exploration. Key features include layout algorithms, styles, and customization options for interactive exploration. Cytoscape supports various network analysis tasks, including topology analysis, clustering, and motif detection, and a network was built. Cytoscape's plugin allows users to extend functionality with additional analysis algorithms, data importers/exporters, and visualization styles. CytoHubba, a popular plugin, focuses on network analysis and identifying important nodes, helping researchers understand key players in biological networks [7]. Cytoscape [7] was used to visualize and analyze biological networks, including drug-gene interactome networks and cytoHubba, a plugin to identify hub genes. These highly connected nodes are crucial for drug response or disease pathways. 

GAT architecture

In this analysis, the PyTorch Geometric library was used to train a GAT model [8,9] for classifying nodes in a graph based on a given dataset. The dataset was prepared by converting certain columns to numeric values and filling any missing values in the node features with zeros. A directed graph was created using NetworkX, with nodes representing entities and edges representing interactions between these entities. The node features and edge indices were converted into tensors suitable for use with PyTorch Geometric. The GAT model consisted of two graph attention layers. The first layer had eight attention heads, each producing eight features, followed by an exponential linear unit (ELU) activation function and dropout. The second layer aggregated the outputs from the first layer into two output features, assuming binary classification. The model was trained using the Adam optimizer with a learning rate of 0.005 and a weight decay of 5e-4, and the CrossEntropyLoss function was used as the loss function.

Evaluation metrics

Evaluation metrics for GAT can be categorized into two main types: node-level metrics and graph-level metrics. These metrics assess the model's performance in predicting node- and graph-level properties, respectively. The model's accuracy measures the overall correctness of predictions, focusing on correctly predicted node properties. Precision measures the proportion of true positive predictions, particularly in classification tasks. Recall measures the proportion of true positive predictions, especially in classification tasks. The F1 score, the harmonic mean of precision and recall, provides a balanced measure of both, allowing for a trade-off between false positives and false negatives.

## Results

The drug-gene interactome network, analyzed using Cytoscape, had 657 nodes, 1591 edges, and 4.755 average neighbors. The network's diameter, radius, and characteristic path length were all measured. The clustering coefficient measured the degree of node connection in groups, while the network density represented the proportion of possible connections. Network heterogeneity measures the variation in connectivity among nodes, while network centralization measures the degree to which the network's connectivity is centered around a few highly connected nodes. The entire network was a single connected component, and the analysis time was 0.396 seconds. These measures offered insights into the network's structure, connectivity, and properties, which could be further analyzed to understand its biological significance and potential drug-gene interactions.

The GAT model's performance in predicting drug-gene interactions for the STAT-1 receptor was influenced by several factors. The initial high training loss values indicated a lack of accuracy, but over time, the model improved its predictions. The receiver operating characteristics (ROC) curve, representing the true positive rate against the false positive rate, indicated that the model's predictions were only slightly better than random chance. Factors such as limited data availability, interaction complexity, and the model's architecture or training approach could have contributed to the model's low accuracy and performance.

**Figure 1:**
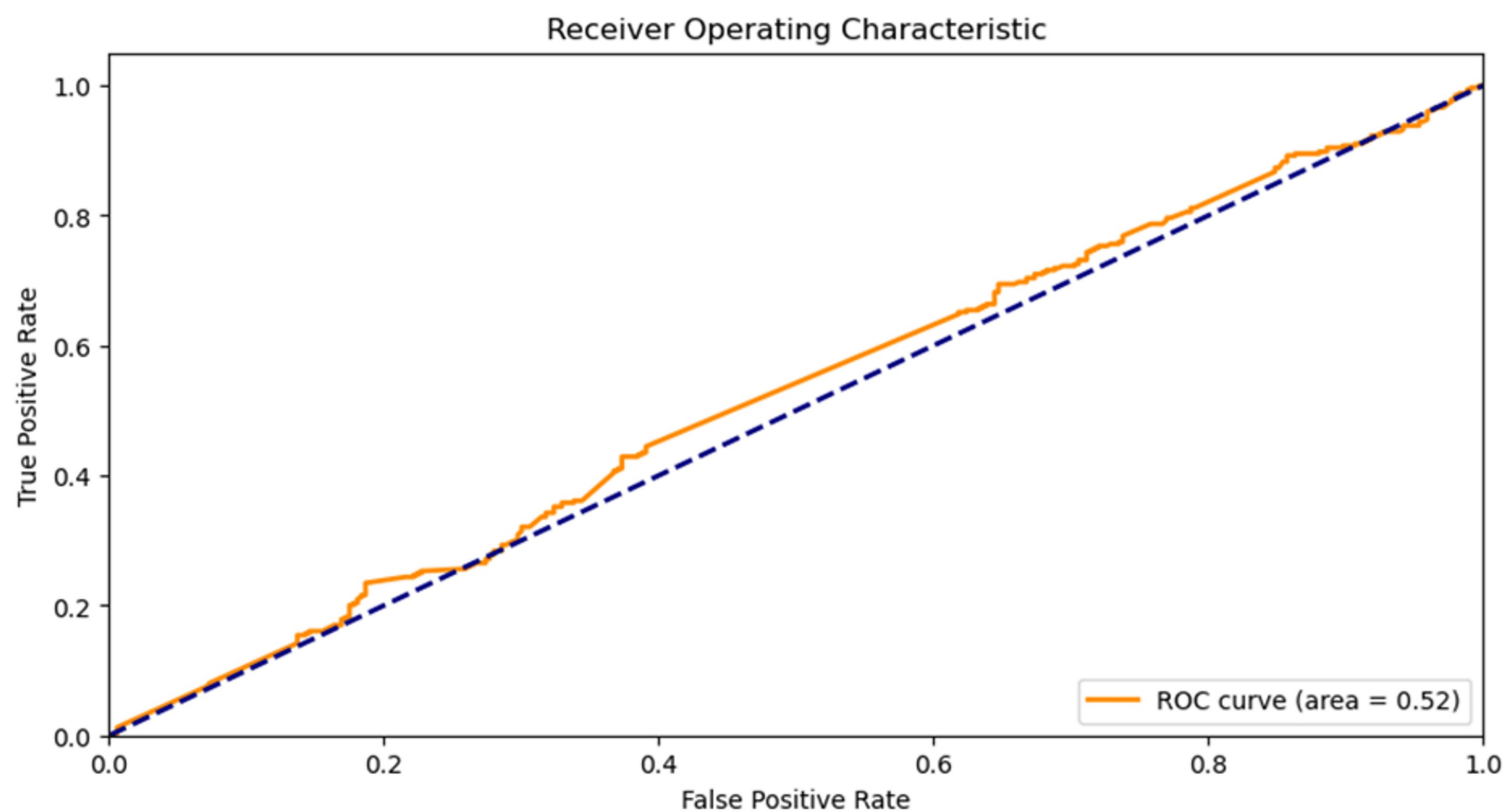
A graph comparing the performance of a binary classifier system with a ROC curve. The ROC curve, a jagged orange line, indicates a performance area of 0.52, slightly above the expected area of random chance. ROC: receiver operating characteristics

The predictive GAT model for drug-gene interactions in the STAT receptor had an accuracy of 0.5, indicating a need for improvement. A graph comparing the performance of a binary classifier system with a ROC curve was made. The ROC curve, a jagged orange line, indicates a performance area of 0.52, slightly above the expected area of random chance, as depicted in Figure 1.

**Figure 2:**
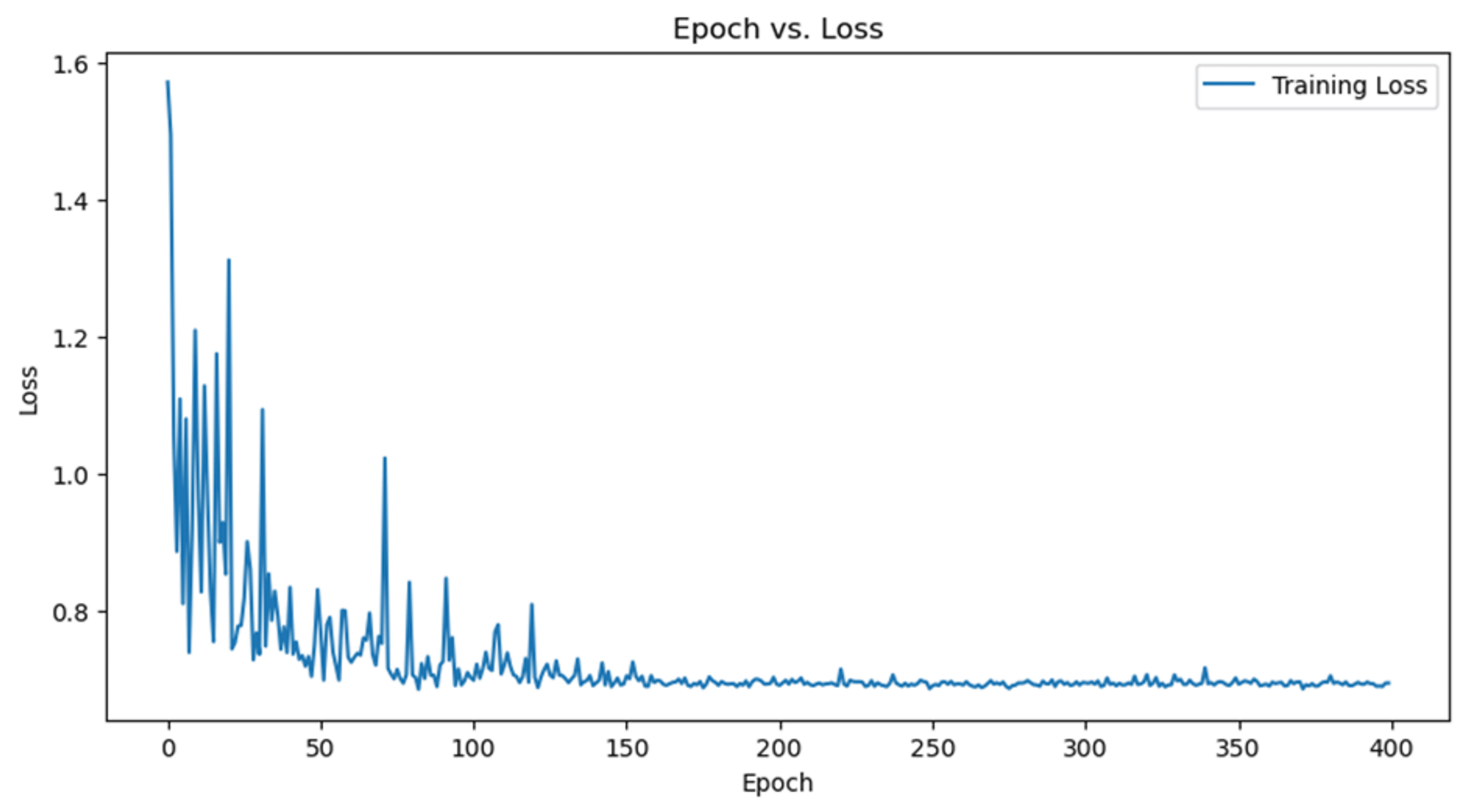
A line graph comparing epochs and loss values during training. The graph shows a "Training Loss" curve with high loss values and a gradual decrease, with results corresponding to the epochs and loss values.

To enhance the accuracy and performance of the GAT model for predicting drug-gene interactions in the context of STAT-1 receptor and periodontal disease, researchers can increase data availability, improve the model's architecture, and focus on feature engineering techniques. This will provide a larger training set, better capture of complex interactions, and enhance the model's ability to accurately predict drug-gene interactions. A line graph comparing epochs and loss values during training was made. The graph shows a "Training Loss" curve with high loss values and a gradual decrease, with results corresponding to the epochs and loss values, as depicted in Figure 2.

## Discussion

The STAT-1 receptor, a member of the STAT family, is crucial in immune responses and cellular processes. It is expressed in immune cells and activates upon binding specific cytokines, mainly IFNs, which play a role in antiviral defense and immune response regulation. The receptor undergoes phosphorylation by JAK, dimerizing and translocating to the nucleus. A previous study explores the link between hypertension and periodontitis using a mouse model. Results show that hypertension increases bone resorption and periodontal destruction, which a specific inhibitor can inhibit. It also increases macrophage infiltration and proinflammatory cytokine expression in periodontitis lesion areas, dependent on the angiotensin II-induced STAT-1 pathway. This leads to the initiation of target gene transcription and downstream effector molecules shaping immune response and cellular behavior [2].

Dysregulated STAT-1 signaling is associated with chronic inflammation, tissue damage, and immune dysfunction. Understanding the STAT-1 receptor's regulatory mechanisms is essential for developing targeted therapies [1].

Machine learning techniques can predict and analyze drug-gene interactions by training models on data about drugs and genes. These techniques can predict new drug-gene interactions or uncover hidden relationships between drugs and genes.

As bioinformatics and chemoinformatics advance, there is increasing focus on complex structured data, such as molecular chemical structures and biological molecular networks like PPIs. These structures are often represented as graphs, with nodes representing atoms or proteins and edges representing chemical bonds or interactions.

Graph neural networks (GNNs) [10,11] have proven effective for analyzing this non-Euclidean data by learning node, edge, or entire graph representations through information propagation and aggregation. Graph convolutional networks (GCNs) assume uniform contributions from neighboring nodes, potentially overlooking intricate interactions. GATs [8,12,13] address this by using attention mechanisms to provide varying weights to different neighbors, particularly relevant in drug discovery. GAT can capture intra-molecular interactions more precisely in a molecule graph, resulting in a more comprehensive and accurate molecular representation. GNN-based [14] drug-drug interactions is a deep learning method for predicting drug-drug interaction-associated events, utilizing drug information from various sources and a deep multi-model framework. It outperforms existing methods, demonstrating the efficiency and effectiveness of producing drug representations based on interaction types and attributes similar to our study results. It shows a lower accuracy of nearly 50 percent in predicting drug-gene interaction of STAT-1 receptors for periodontal inflammation using GATs.

Limitations

The predictive GAT model [15,16] for drug-gene interactions in the STAT receptor is criticized for its low accuracy in periodontal inflammation. Factors such as limited data availability, interaction complexity, and inability to capture all relevant features may limit its effectiveness. Larger and more comprehensive datasets are needed to improve the model's performance. The model may not capture all relevant features, such as gene expression levels or post-translational modifications, which could be improved by incorporating additional features. Future directions for improving drug-gene interaction predictions in periodontal inflammation include data collection and annotation, model refinement or development, integration of complementary data sources, and validation and experimental validation. Addressing these limitations can enhance the accuracy of predictive models for drug-gene interactions in the STAT receptor context. Collaboration between bioinformatics, computational biology, and experimental researchers is crucial for advancing this field and improving the prediction and understanding of drug-gene interactions in periodontal inflammation [5,17].

## Conclusions

The GAT model's current architecture may not fully capture the complexity of drug-gene interactions in periodontal inflammation, leading to lower accuracy rates. To improve accuracy, researchers should refine the model's architecture and training approach, incorporate domain-specific knowledge, and incorporate complementary data sources like genomics, proteomics, and metabolomics. Validating predictions through experimental and clinical data is crucial for assessing the model's reliability in real-world scenarios. Addressing limitations of data availability, interaction complexity, and inability to capture all relevant features can help develop effective treatments for periodontal inflammation.
